# Biotelemetry marches on: A cost-effective GPS device for monitoring terrestrial wildlife

**DOI:** 10.1371/journal.pone.0199617

**Published:** 2018-07-31

**Authors:** Manuela Fischer, Kate Parkins, Kean Maizels, Duncan R. Sutherland, Blake M. Allan, Graeme Coulson, Julian Di Stefano

**Affiliations:** 1 School of Ecosystem and Forest Sciences, University of Melbourne, Creswick, VIC, Australia; 2 Conservation Department, Phillip Island Nature Parks, Cowes, VIC, Australia; 3 Kean Electronics, Mount Kuring-Gai, NSW, Australia; 4 School of Life and Environmental Sciences, Deakin University, Burwood, VIC, Australia; 5 Department of BioSciences, University of Melbourne, Parkville, VIC, Australia; University of Sydney, AUSTRALIA

## Abstract

The availability of low-cost wildlife trackers increases the capacity to collect valuable ecological data when research budgets are limited. We converted a commercially available global positioning system (GPS) product into a low-cost tracking device that sends data via the mobile phone network, and assessed its performance under varying conditions. We established a stationary test, deploying devices along a continuum from open urban areas to topographically and structurally complex forested sites. We tested three features of the device: (a) the GPS, by measuring fix success rate, fix precision and horizontal dilution of precision (HDOP), (b) remote download capacity via the mobile phone network and (c) battery drain. Measures of GPS performance demonstrated high fix success rates and precision. HDOP values were influenced by habitat type and topographical position, but generally remained very low, giving an acceptable degree of error for most applications in wildlife research. Devices experienced delayed data transmission at sites with less phone reception, and faster battery drain at sites with denser vegetation. We recorded device malfunctions in 8.2% of the 110 sampling locations, but these were not associated with habitat type or topography. Our device was effective under a wide range of conditions, and the development process we used provides guidance to other researchers aiming to develop cost-effective wildlife trackers. Reducing the financial and labour costs of acquiring high-quality movement data will improve the capacity to increase sample size in animal movement studies.

## Introduction

Animal movement is a key process influencing survival and reproduction [1, 2]. Movement is fundamental to behaviours such as acquiring food, shelter and mates, avoiding predators, and dispersing to new habitats [1]. Further, understanding how animals move within and between landscapes will provide a sound basis to improve management. However, data are limited for many species and data collection remains a major challenge [2].

Spatial ecologists commonly employ the Global Positioning System (GPS) to acquire movement data. The advantages of GPS technology include increased data quantity and quality, and reduced labour time and costs. The latter can be reduced further by coupling GPS with the mobile phone network which eliminates the need to extract the data via a local radio-link, or to recapture the tagged animal and download stored data, as the data can be collected remotely.

Although these new technologies have numerous benefits and have made significant improvements to the quality and quantity of data researchers can obtain, commercially available tracking devices remain expensive. As a result, researchers often purchase a small number of units [3], reducing sample size and limiting population-scale inference [4–7].

A potential solution is to purchase off-the shelf GPS components and modify them for wildlife trackers. Although this strategy has been successfully applied on several occasions [4, 5, 7], it is not widespread. Moreover, using remote data transmission via the mobile phone network has been successful in some studies [8, 9] but implementing this technology into custom-made wildlife trackers is rare. For example, Quaglietta et al. [10] developed and tested a GPS wildlife tracker linked to the phone network for otters (*Lutra lutra*) and reported a reliable method of GPS telemetry in riparian and aquatic environments. By combining a data-logging function with a remote download feature, data loss is minimised, as recapturing animals to regain data is often difficult or local download restricted (i.e. home ranges are large or located in remote or challenging environments). As far as we are aware, no studies have tested custom-made wildlife trackers with remote download function in a range of terrestrial environments and under varying environmental conditions.

In this study, we (a) describe the development of a custom-made GPS/High Speed Packet Access (HSPA) wildlife tracker and (b) determine the performance of our devices by testing GPS function, remote download capacity and battery drain rate along a continuum from urban areas to topographically complex forested sites.

We chose two study sites, that differ in vegetation cover, topographical levels and phone network coverage to test the performance of our trackers. We expected varying results between study locations but demonstrate that both GPS and remote download capacity performed very well under a range of conditions.

## Methods

### GPS wildlife tracking system development

We purchased MT-900C tracking devices from UniTraQ (UniTraQ International Corp., Taiwan) at a unit cost of US$175. Unlike many low-cost tracking devices, the MT-900C combines a GPS and 3G HSPA mobile interface, and can be configured to store GPS fixes in an internal 4 MB flash memory (up to 944 records). Fixes are stored on the device until the next active phone network connection, when data are sent via text message or to a specific IP address via a HSPA internet connection. In addition to GPS fix (reported as longitude and latitude), each record delivers information including speed (m/s), send/resend status, battery status (mV) and cellular signal quality (CSQ).

We modified the firmware to increase battery life and included several additional variables in each record. We decreased power consumption by reducing the duration of the awake mode in the awake–sleep cycle between fix attempts. Further, we programmed the device to report altitude, number of satellites and horizontal dilution of precision (HDOP) to help define the quality of each fix. Secondly, we made changes to the hardware by extending the GPS antenna, enabling this to point towards the sky when the device was built into a collar, increasing the likelihood of locating satellites [11]. To extend battery life, we replaced the original battery with a pair of 3.6-V lithium ion battery cells (Panasonic NCA103450 2350mAh, Master Instruments Pty Ltd., NSW, Australia), which required the attachment of an external battery connector to the circuit board.

We used Plasticast, a fast-curing, two-component, rigid, urethane casting compound (Dalchem Pty Ltd., VIC, Australia; Barnes Products Pty Ltd., NSW, Australia) to embed the tracker components in a shock resistant and waterproof casing. We built the casing using a mould designed to fit the tracker components and to suit our study species, the swamp wallaby (*Wallabia bicolor*). We embedded the batteries and device circuit board (including the 3G antenna) within the casing and sealed the lid using silicon. We attached a 25 mm wide synthetic webbing tube to the casing to function as a collar. The GPS antenna, embedded in epoxy putty (Selleys Pty Ltd, NSW, Australia) and covered with silicon spray, was fitted within the collar webbing opposite the casing. The antenna wire was positioned inside the tube to prevent damage. A video showing how collars were built is available at S1 Video.

Finally, we developed an internet server to facilitate communication with deployed devices, data download, and initial data visualisation by displaying fixes in Google Maps. The cost of each unit, including firmware change and development of the server, was US$366.81 (see detailed list of costs in S1 Table). The weight of the custom-made tracker was 180g.

### Stationary field test

#### Study locations

**Fig 1:**
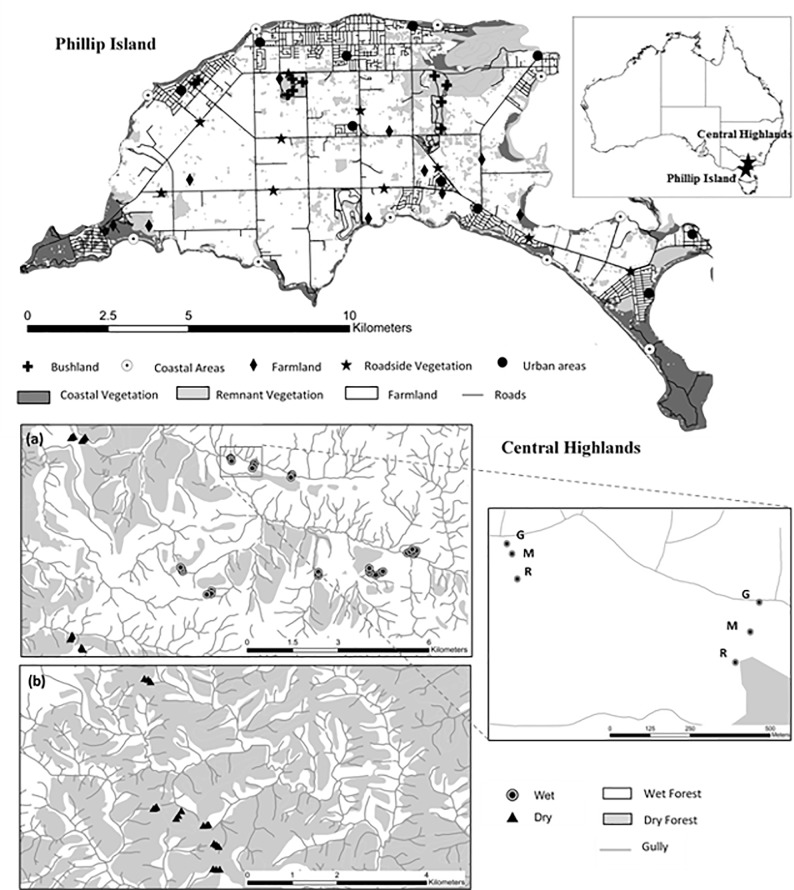
Location of the two study areas, Phillip Island and the Central Highlands. Sites in the Central Highlands are positioned in blocks of three; gully (G), mid-slope (M) and ridge (R). Locations (a) and (b) represent the two spatially separated study sites of the Central Highlands.

In a stationary test, we systematically tested the devices at two locations, Phillip Island and the Victorian Central Highlands, to represent a range of conditions likely to be encountered in animal GPS tracking studies. The two locations differed with respect to vegetation cover, topographical complexity and phone network coverage (Fig 1).

Phillip Island (38° 29’ S; 145° 15’ E) is a 100-km^2^ landmass 90 km south-east of Melbourne, Australia. Relief is mostly low, with a maximum altitude of 112 m above sea level. The landscape contains patches of agricultural land, coastal and roadside vegetation, urban centres and small (<100 ha) patches of native bushland (Fig 1).

The Victorian Central Highlands (37° 75’ S; 145° 69’ E) ranges in elevation from 200–1500 m above sea level. The landscape is topographically complex with much of the region characterised by steep (>20°) slopes and gullies. We selected two sub-regions within the Central Highlands study area differing in canopy cover and vegetation complexity: wet and dry forest. Wet forest was characterised by a tall *Eucalyptus regnans* or *E*. *delegatensis* overstorey, with a tall broad-leaved shrubby understorey and a moist, fern-rich ground layer. Dry forest was dominated by messmate stringybark (*E*. *obliqua*) and broad and narrow-leaf peppermints (*E*. *dives* and *E*. *radiata*), and a diverse open understorey of shrubs, grasses and herbs. Dry forest was characterised by a more open canopy than wet forest (Fig 1).

#### Study design

We conducted systematic tests of the devices between February and May 2016. On Phillip Island, we chose five different habitat types to represent the range of terrestrial environments present: remnant bushland, farmland, roadside vegetation and urban and coastal areas. In each habitat type, we randomly selected ten sites using ArcGIS 10.2.1 [12], giving 50 sites in total (Fig 1). The minimum distance from the edge of each habitat type was 25 m and minimum distance between each sampling site was 100 m.

We initially stratified the Central Highlands study area into wet forest and dry forest. In each forest type, we distributed sites in clusters of three (gully, mid-slope and ridge), with randomly chosen sites in gullies defining the position of the other two sites in the cluster (Fig 1). Clusters were at least 100 m apart, while sites within clusters were separated by a minimum distance of 30 m. We excluded areas of recent disturbance (<10 years), such as fire or logging from the sampling area. There were 10 clusters (30 sites) in each forest type, giving 60 sites in total.

We used ten different devices on Phillip Island and the same ten plus an additional five in the Central Highlands. We positioned each randomly-chosen device on the ground at each site and secured it to a wooden stake with adhesive tape. Devices were scheduled to record fixes every 30 minutes, a rate used in many studies [9, 13, 14]. Devices remained at each site for a period of approximately 24 hours, before being moved to another site.

#### Data analysis

We selected three response variables to test GPS performance: fix success rate, fix precision and horizontal dilution of precision (HDOP). We assessed remote download capacity using a variable representing the cellular signal strength (CSQ), and assessed battery drain using the reduction in voltage over the deployment period standardised by the number of fix attempts. As predictor variables, we chose habitat type (both study areas), topographic position (Central Highlands) and, in the case of remote download and battery drain, the CSQ value, as these factors were expected to influence the performance of the device. For some analyses we also included the number of successful fixes as a continuous predictor. Because topography was relevant only in the Central Highlands, we analysed each response variable separately in each study area. Details of response and predictor variables are presented in Table 1. Malfunctioning devices with less than two successful fixes (n = 6; 5.5%), and fixes with no coordinates and HDOP and CSQ values of 99, were excluded from the analysis (Phillip Island: n = 806; 16.9%; Central Highlands: n = 2630, 22.9%).

**Table 1:** Overview of all variables.

Response variable	Description	Predictor variables
*GPS performance*		
**Fix success (%)**	Percentage of successful fixes (number of fixes with X and Y coordinates divided by the number of fix attempts)	Habitat Type
		Topography (CH)
**Fix precision**	The median distance in meters between each GPS fix and the median fix location.	Habitat Type
		Topography (CH)
		Number of fixes
**Horizontal dilution of precision (HDOP)**	Geometric quality of the satellites contributing to the GPS position^1^	Habitat Type
		Topography (CH)
		Number of fixes (CH)
*Remote download capacity*		
**Data transmission (%)**	Percentage of fixes sent from the device with no temporal delay	Habitat Type
		Topography (CH)
		Number of fixes
		Cellular signal quality^2^
*Battery drain*		
**Voltage loss index**	mV used during deployment divided by the number of fix attempts	Habitat Type
		Topography (CH)
		Cellular signal quality^2^

Response and predictor variables used for testing the devices’ (a) GPS performance, (b) remote download capacity via the phone network (c) battery drain rate. Predictor variables followed by (CH) were only used to analyse data from the Central Highlands study area. Habitat types were bushland, farmland, roadside vegetation, urban and coastal areas on Phillip Island and wet and dry forest in the Central Highlands. Topography was classified as a three-level variable representing gully, mid-slope and ridge.

^1^ Values can range from < 1 (ideal) to > 20 (poor).

^2^ Cellular signal quality (CSQ) represents the signal strength of the mobile network and ranges from 0 (no signal) to 31 (high signal strength).

Two of the response variables, fix success rate and data transmission rate (Table 1), were not amenable to formal analysis due to their limited range: most of the values for both variables were 100%. However, graphical data exploration revealed a strong, non-linear relationship between data transmission and phone signal strength (CSQ), so we displayed this using a scatter plot and smoothing spline.

We determined associations between the remaining three response variables and their predictors (Table 1) using either linear models (LM) or linear mixed models (LMM) with Gaussian error distributions, having tested assumptions of normality, independency and homogeneity of variance. For each response variable separately, we initially compared a global fixed model with and without device ID as a random factor, accepting the form of model with the lowest AICc (Akaike’s Information Criterion corrected for small sample size) for subsequent analysis [15]. Using this procedure, we included device ID as a random factor and used a LMM to analyse HDOP on Phillip Island and battery drain in the Central Highlands. We ran all other models as simple linear models with no random factor. We built models in the R statistical environment [16] using the package nlme [17].

For each response variable in each study area, we produced a candidate set of models consisting of each individual predictor variable and variables in additive and interactive combinations. The size of the candidate set in each case was defined by the number of predictors, which differed among response variables and study areas (Table 2). Within each candidate model set, we used AIC_c_ and Akaike weights to indicate the degree of support for each model [18]. We assessed model fit using R^2^. For LMM, we used the method of Nakagawa et. al [19], which yields marginal R^2^ (R^2^m), the variance explained by fixed factors, and conditional R^2^ (R^2^c), the variance explained by both fixed and random factors. We conducted model selection and the R^2^ calculation for mixed models using the package MuMIn [19, 20].

**Table 2:** Full interaction model structure of all predictor variables.

Response Variable	Global fixed model	Random effect structure
***(a) Phillip Island***		
HDOP	HT	Device ID
Fix precision	HT x Fix	None
Battery drain	HT x CSQ	None
***(b) Central Highlands***		
HDOP	HT x Fix x Topo	None
Fix precision	HT x Fix x Topo	None
Battery drain	HT x CSQ x Topo	Device ID

(*a*) Phillip Island and (*b*) the Central Highlands. Habitat type (HT), Number of fixes taken (Fix), Topography (Topo), phone signal strength (CSQ) and the random variable (Device ID) after testing the significance of the random factor in the model. On Phillip Island the number of fixes taken for HDOP was uneven among HT and omitted.

## Results

**Fig 2:**
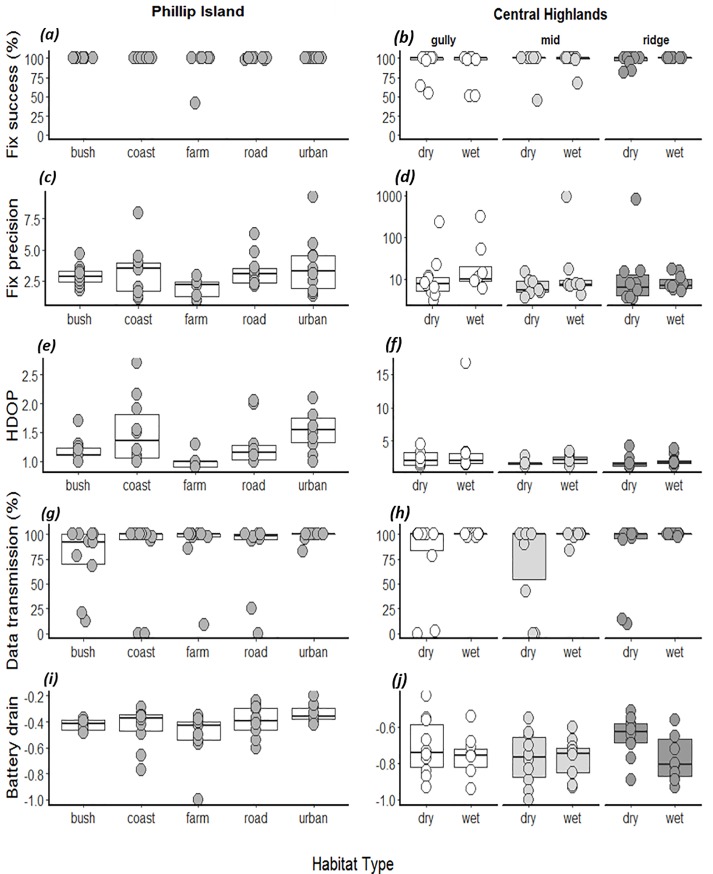
Raw data of all variables. *(a) and (b)* fix success (%), (*c*) and (*d*) fix precision, *(e)* and *(f)* Horizontal dilution of precision (HDOP), (*g)* and *(h)* data transmission (%) and (*i*) and (*j*) battery plotted against habitat type and, for the Central Highlands, topography (gully, mid-slope and ridge). Boxplots indicate median and first and third quartile. To improve visual display dots of (*a*), (*b*), (*d*), (*g*) and (*h*) have been jittered and data of (*d*) has been log10 transformed.

**Fig 3:**
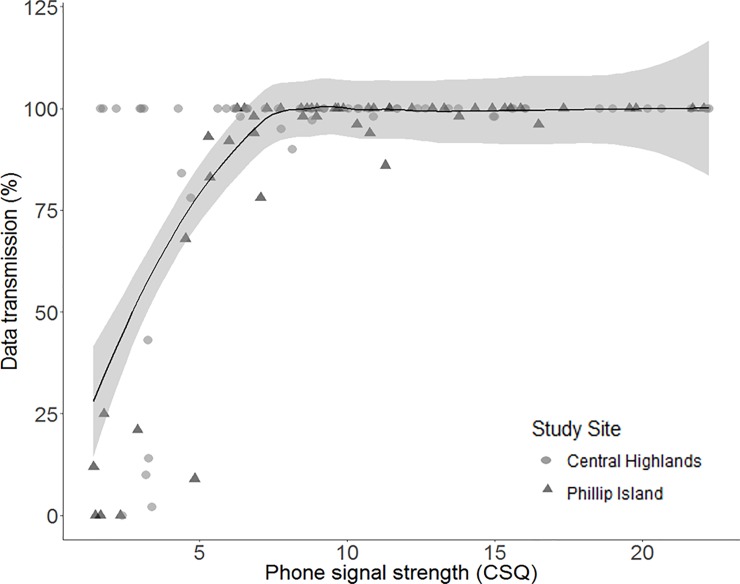
Data transmission. Percentage of GPS fixes sent immediately through the phone network plotted against phone signal strength (CSQ) combined for both study sites. Black line represents a locally weighted smoothing spline with 95% confidence intervals (grey shading).

**Fig 4:**
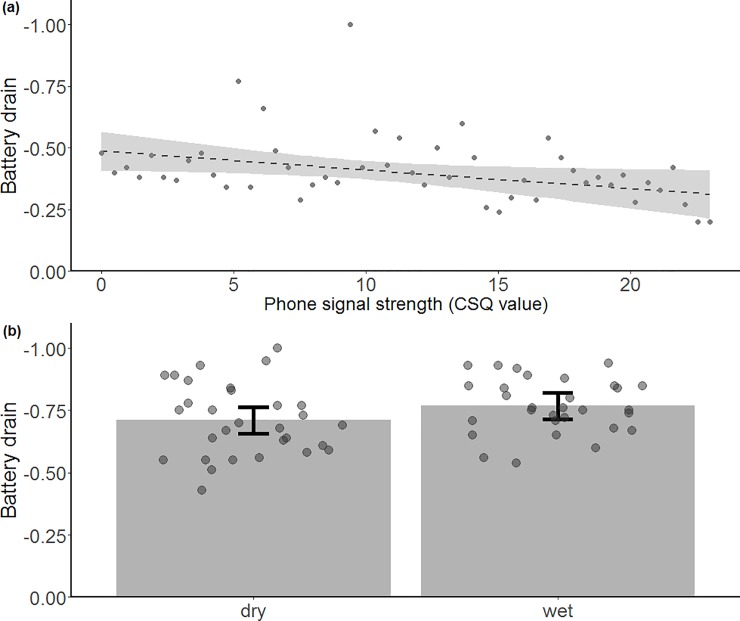
Battery drain rate. Battery drain rate for each sample location in *(a)* Phillip Island plotted against phone signal strength (CSQ) and *(b)* Central Highlands represented in wet and dry forest. Values closer to 0 indicate less battery drain. Predictions are from linear models and grey dots represent raw data, jittered in (*b*). Shaded areas and error bars indicate 95% confidence intervals.

Raw data for each of the five response variables in each study site are shown in Fig 2. Fix success rate was 100% in 93.9% of the sampling locations on Phillip Island and in 76.4% of the locations in the Central Highlands. On Phillip Island, the median fix precision (±95% CI) in all habitat types combined was good (2.83 ± 0.48 m) and better than in the Central Highlands (7.52 ± 47.01 m). Median HDOP values (±95% CI) were very low (good) on Phillip Island (1.20 ± 0.11) and the Central Highlands (1.70 ± 0.58), respectively (Fig 2). Immediate data transmission was high in both study sites (Figs 2 and 3) and battery drain (±95% CI) was higher in the Central Highlands (-0.75 ± 0.03) compared to Phillip Island (-0.39 ± 0.04) (Figs 2 and 4).

**Fig 5:**
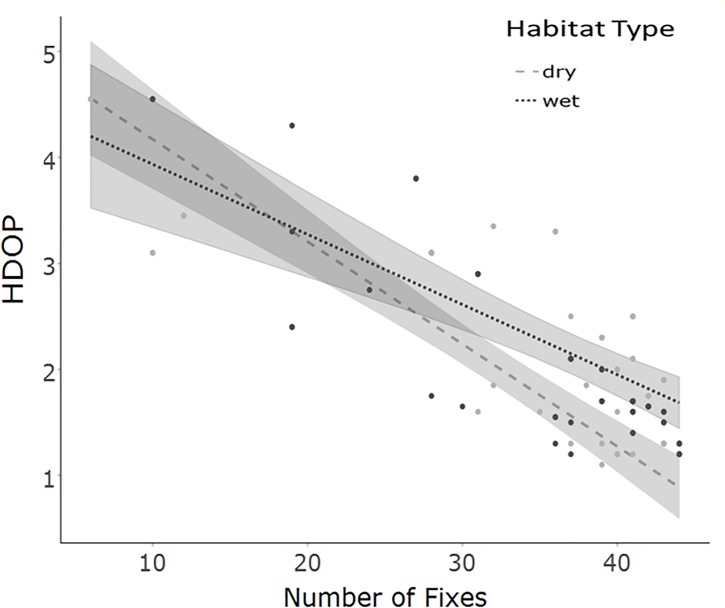
Response of horizontal dilution of precision (HDOP). HDOP values in different habitat types and topographic levels are plotted against number of fixes in the Central Highlands. Predictions are from linear models and dots represent raw data. Shaded areas indicate 95% confidence interval.

Statistical modelling suggested that fix precision was not influenced by any of the predictor variables in either study location (Table 3A and 3B). On Phillip Island, HDOP was influenced by habitat type (Table 3A). However, HDOP values for all habitat types were very low (Fig 5), indicating similar GPS fix quality across the island. In the Central Highlands, there was an interaction between habitat type and the number of fixes (Table 3B): devices that collected fewer fixes had higher HDOP values, but this effect was more pronounced in dry forests than wet forests (Fig 5). Nevertheless, HDOP values were low in both habitat types and across the number of fixes taken.

**Table 3:** Table of model responses.

Variable	Model sets	ΔAIC_c_	Akaike weight	R^2^m (R^2^c)
***(a) Phillip Island***				
HDOP	HT	0.00	0.953	0.236 (0.461)
	NULL	6.02	0.047	0.000 (0.189)
Fix precision	NULL	0.00	0.634	0.000
Battery drain	CSQ	0.00	0.539	0.100
	NULL	2.90	0.127	
***(b) Central Highlands***				
HDOP	HT x Fix	0.00	0.743	0.565
	NULL	40.53	0.000	
	HT x Topo	45.90	0.000	0.099
Fix precision	NULL	0.00	0.644	0.000
Battery drain	HT	0.00	0.274	0.051 (0.188)

Responses of horizontal dilution of precision (HDOP), precision and battery drain to habitat type (HT), Number of fixes taken (Fix), Topography (Topo) and phone signal strength (CSQ) on (*a*) Phillip Island and in the (*b*) Central Highlands. Akaike’s information criteria adjusted for small sample sizes (AIC_c_) was used to rank models. Models within two AIC_c_ units of the top ranked models and the null model are shown in Akaike weights (Aw) and are displayed with two measures of fit: *R*^*2*^m and *R*^*2*^*c*. *(b)* HDOP includes top ranked model, null model and least supported model.

The capacity of devices to transmit data immediately via the phone network was generally high, but reduced substantially at very low network signal strength (Fig 3). Most fixes were sent immediately when CSQ values were greater than 7.5 (n = 65). Of the 65 devices with CSQ > 7.5, 80% recorded the transmission of all fixes taken with no delay. Nevertheless, the devices still performed well in areas with CSQ values ≥ 5 (n = 82), where the immediate data transmission rate was > 75%. The remaining stored data was transmitted to the server when the next successful phone network connection was established, leading to no data loss overall.

The only variable influencing battery drain on Phillip Island was cell signal strength (Fig 4A). In contrast, battery drain was influenced by habitat type in the Central Highlands, with batteries draining faster in wet than dry forests (Fig 4B). However, the *R*^*2*^m value of this model was low (*R*^*2*^m = 0.051) and the AIC_c_ value was similar to the null model (ΔAIC_c_), both indicating the model to be a poor fit to the data.

Our devices failed 8.2% of the time (9 of 110 sites), with 7 of the 9 failures in the Central Highlands (Table 4). Failures did not appear to be associated with particular devices, habitat types or topographic positions, although the data were too sparse to analyse this formally. Characteristics of each failure are described in Table 4.

**Table 4:** Listing of malfunctioning GPS devices.

Habitat Type	Topography	Device ID	Fix attempts	Successful fixes (in %)^1^	Fixes with HDOP failure (in %)	Comments
***(a) Phillip Island***						
**Farmland**	NA	8	22	100	9	Device stopped taking fixes after 22 successful fix attempts.
**Farmland**	NA	9	47	0	100	No successful fixes taken
***(b) Central Highlands***						
**Wet**	Gully	2	43	47	93	Low fix quality
**Wet**	Gully	3	41	54	100	Low fix quality
**Wet**	Mid	9	42	0	100	No successful fixes taken
**Wet**	Mid	10	41	7	100	Low fix quality
**Dry**	Gully	4	40	100	85	Low fix quality
**Dry**	Mid	1	40	0	100	No successful fixes taken
**Dry**	Mid	6	37	92	100	Low fix quality

Numbers of total successful fixes compared to fix attempts and the number of fixes with HDOP errors.

^1^ Fixes with X and Y coordinates

## Discussion

In spatial ecology, using GPS telemetry devices is a common tool to acquire animal movement data. Although commercially available systems are effective, they are costly and therefore unavailable for projects with budget limitations. In this study, we designed and built cost-effective wildlife trackers and examined their performance in a range of terrestrial environments likely to be encountered by researchers using terrestrial GPS tracking technologies.

### Building GPS wildlife trackers

Building wildlife trackers proved to be very cost efficient, saving around 80% of the regular costs for commercially available devices with similar functions. Allan et al. [5], for example, highlights prices of several GPS-based devices which could have been used to build a wildlife tracker and are similar in cost to ours. Although it requires time and therefore cost to design, build and test the trackers (S1 Table), new technologies such as 3D printers to form the tracker casing could be used in the future to reduce some of these expenses. Further, custom building wildlife trackers provides the ability to specifically adjust the design of the tracker and the devices’ firmware to better suit the study species and address research questions [5].

### Stationary field test

*GPS performance*: We expected poorer performance from the device in areas with denser vegetation and more complex topography. However, the GPS functioned well under a wide range of conditions. Fix precision in both of our study areas and along a range of habitats and topographic levels was high and none of the predictor variables tested influenced precision. Although our model outputs showed that HDOP values were influenced by some predictor variables (habitat type and number of fixes taken), the majority of these values were low, suggesting that none of our predictor variables had an appreciable impact on precision. D'Eon et al. [21] and D'Eon et al. [22] found that fix precision and HDOP values depended on the amount of available sky, and that animals inhabiting topographically flat and open areas receive GPS locations of better quality than animals on slopes and within denser vegetation. In our study, the HDOP values in the Central Highlands increased slightly when the numbers of fixes were low and sites were more vegetated (wet forest), yet they did not exceed the range representing good fix quality, which might be due to recent technical improvements.

*Phone Network*: Although the success rate of the remote data transmission was generally high, areas with very weak phone network connection experienced delays in data transmission (Fig 3). Our results suggest that immediate data transmission will usually occur if study areas have CSQ values greater than 5. However, the remote download capacity may fail completely in more remote areas where the phone network connection is very poor or unavailable. However, this could be improved through the use of a wireless-sensor network that does not require telecommunication support, as trialled by Juang et al. [23]. Other options to download data remotely in areas with patchy or no phone receptions are satellite-linked GPS wildlife trackers such as GPS-Iridium or GPS-Globalstar, but purchasing them commercially remains expensive [3, 20, 24]. However, Lehrke et al. [25] used off-the-shelf GPS-Globalstar trackers and successfully tracked black swans (*Cygnus atratus*) in New Zealand for a fraction of the cost of commercial units.

*Battery drain*: Battery drain increased with decreasing network strength on Phillip Island and was higher in wet forest than dry forest in the Central Highlands. If fixes are taken every 15 minutes, as it was the case in the collar-mounted devices, battery life was less than 10 days using two 3.6-V lithium ion batteries. In general, frequent remote downloads significantly increase power consumption, having an impact on battery life, especially in areas of poor reception when the device continues searching for phone network connection (Fig 4). Thus, researchers face a trade-off: they can use devices with a remote download function, but face high battery drain rates, or they can download the data in the field using an antenna to download data by recapturing animals, or with functions such as self-release mechanisms [26, 27]. Downloading in the field, however, will increase labour time and most likely costs. Yet, a major advantage of custom-made trackers is that the batteries of recovered collars can easily be recharged and redeployed, avoiding the additional costs required to refurbish most commercial trackers. To increase the life of the battery, solar powered or energy harvesting GPS trackers, or increased battery capacity, could be considered as future improvements to our device. The firmware could also be improved in a number of ways to better manage battery life, such as using CSQ values below a certain threshold to selectively store the fix data for later transmission, or requesting fewer transmission attempts.

Despite a generally good performance of the stationary devices we also experienced occasional malfunctions (Table 2). The main issues were errors in HDOP estimation, leading to fixes lacking X, Y coordinates or fixes with low fix quality. Further, one device stopped working completely. Currently we have no explanation for these malfunctions; faulty devices worked well again when reset and retested in other sampling locations.

Finally, we note that animal behaviours that result in poor orientation of the GPS antenna or increase the instance of physical barriers between the antenna and the satellites may reduce GPS performance, but are not accounted for in stationary tests. To ensure stationary devices provide a rigorous test of new GPS products, spatially matched data from stationary and animal-born devices should be similar, but to our knowledge this comparison has never been made. Although it was beyond the scope of this study to comprehensively compare data from stationary and animal-born devices, we conducted a pilot project using data from two swamp wallabies (*Wallabia bicolor*) moving within a 25 ha grid of 100 stationary devices. We present the pilot project in S1 Appendix. Our intention is to highlight the need for further research, and to provide some initial methods for data analysis.

## Conclusion

Neither habitat type nor topography had a major impact on the performance of the GPS, remote download capacity or battery, indicating that our device will function well in a wide range of habitats with adequate phone network connection. Although phone signal strength influenced battery drain rate, likely resulting in shorter battery life in areas of low or patchy phone reception, an advantage of having remote download capacity is that researchers reduce valuable labour time in the field acquiring the data. Finally, a major advantage of our device is its low cost. In many situations this will enable larger samples sizes, improving the capacity to make population-level inferences, and to contrast behaviour among different habitat types or demographic classes.

## Supporting information

S1 Table GPS wildlife tracker costs. Total cost of GPS wildlife tracker per unit excluding postage.
(DOCX)

S1 Video Development of a wildlife tracker. (MP4)

S1 Appendix Comparing spatially-matched GPS data from stationary and animal-born devices. (DOCX)

S1 Dataset Dataset underlying the findings described for the Central Highlands analysis (CSQ/battery drain). (CSV)

S2 Dataset Dataset underlying the findings described for the Central Highlands analysis (HDOP). (CSV)

S3 Dataset Dataset underlying the findings described for Phillip Island analysis (CSQ/battery drain). (CSV)

S4 Dataset Dataset underlying the findings described for Phillip Island analysis (HDOP). (CSV)

## References

[1] Fahrig L. Non-optimal animal movement in human-altered landscapes. Funct Ecol. 2007;21(6):1003–15. 10.1111/j.1365-2435.2007.01326.x PubMed PMID: WOS:000250985100001.

[2] Nathan R, Getz WM, Revilla E, Holyoak M, Kadmon R, Saltz D, et al A movement ecology paradigm for unifying organismal movement research. Proc Natl Acad Sci U S A. 2008;105(49):19052–9. 10.1073/pnas.0800375105 ; PubMed Central PMCID: PMC2614714. 19060196 PMC2614714

[3] Thomas B, Holland JD, Minot EO. Wildlife tracking technology options and cost considerations. Wildlife Research. 2011;38(8):653–63. 10.1071/WR10211.

[4] Zucco CA, Mourão G. Low-cost global positioning system harness for pampas deer. Journal of Wildlife Management. 2009;73(3):452–7. 10.2193/2007-492

[5] Allan BM, Arnould JPY, Martin JK, Ritchie EG. A cost-effective and informative method of GPS tracking wildlife. Wildlife Research. 2013;40(5):345–8. 10.1071/wr13069 PubMed PMID: WOS:000324869700001.

[6] Forin-Wiart MA, Hubert P, Sirguey P, Poulle ML. Performance and Accuracy of Lightweight and Low-Cost GPS Data Loggers According to Antenna Positions, Fix Intervals, Habitats and Animal Movements. PLoS One. 2015;10(6):e0129271 10.1371/journal.pone.0129271 ; PubMed Central PMCID: PMC4472960. 26086958 PMC4472960

[7] Clark PE, Johnson DE, Kniep MA, Jermann P, Huttash B, Wood A, et al An Advanced, Low-Cost, GPS-Based Animal Tracking System. Rangel Ecol Manag. 2006;59:334–40.

[8] Rahimi S, Owen-Smith N. Movement patterns of sable antelope in the Kruger National Park from GPS / GSM collars: a preliminary assessment. South African Journal of Wildlife Research. 2007;37(2):143–51.

[9] Dettki H, Ericsson G, Edenius L. Real-time moose tracking: An internet based mapping application using GPS/GSM collars in Sweden. ALCES. 2004;40:13–21.

[10] Quaglietta L, Martins BH, de Jongh A, Mira A, Boitani L. A low-cost GPS GSM/GPRS telemetry system: performance in stationary field tests and preliminary data on wild otters (*Lutra lutra*). PLoS One. 2012;7(1):1–10. 10.1371/journal.pone.0029235 ; PubMed Central PMCID: PMC3252312. 22242163 PMC3252312

[11] Belant JL. Effects of antenna orientation and vegetation on global telemetry systems. Northeastern Naturalist. 2009;16(4):577–84. 10.1656/045.016.n407

[12] Esri AD. Release 10. Documentation manual Redlands, CA, Environmental Systems Research Institute. 2011.

[13] Pépin D, Adrados C, Mann C, Janeau G. Assessing real daily distance traveled by ungulates using differential GPS locations. Journal of Mammalogy. 2004;85(4):774–80.

[14] Benhamou S. Dynamic approach to space and habitat use based on biased random bridges. PloS one. 2011;6(1):e14592 10.1371/journal.pone.0014592 21297869 PMC3027622

[15] Zuur A, Ieno E, Walker N, Saveliev A, Smith G. Mixed effects models and extensions in ecology with R New York: Springer 2009.

[16] R Core Team. R: A language and environment for statistical computing. R Foundation for Statistical Computing. Vienna, Austria2014.

[17] Pinheiro J, Bates D, DebRoy S, Sarkar D, Heisterkamp S, Van Willigen B, et al. Package ‘nlme’. 2016.

[18] Burnham KP, Anderson DR. Model selection and multimodel inference: a practical information-theoretic approach: Springer Science & Business Media; 2002.

[19] Nakagawa S, Schielzeth H, O'Hara RB. A general and simple method for obtaining R2 from generalized linear mixed-effects models. Methods in Ecology and Evolution. 2013;4(2):133–42. 10.1111/j.2041-210x.2012.00261.x

[20] Bartoń K. MuMIn: multi-model inference. R package version. 2013.

[21] D'Eon RG, Serrouya R, Smith G, Kochanny CO. GPS radiotelemetry Error and Bias in Mountain Terrain. Wildlife Society Bulletin. 2002;30(2):430–9.

[22] D'Eon RG, Delparte D. Effects of radio-collar position and orientation on GPS radio-collar performance, and the implications of PDOP in data screening. Journal of Applied Ecology. 2005;42(2):383–8. 10.1111/j.1365-2664.2005.01010.x

[23] Juang P, Oki H, Wang Y, Martonosi M, Peh LS, Rubenstein D, editors. Energy-efficient computing for wildlife tracking: Design tradeoffs and early experiences with ZebraNet. ACM Sigplan Notices; 2002: ACM.

[24] Tomkiewicz SM, Fuller MR, Kie JG, Bates KK. Global positioning system and associated technologies in animal behaviour and ecological research. Philos Trans R Soc Lond B Biol Sci. 2010;365(1550):2163–76. 10.1098/rstb.2010.0090 ; PubMed Central PMCID: PMC2894966. 20566494 PMC2894966

[25] Lehrke RM, McGregor L, Dyer J, Stanley MC, Dennis TE. An inexpensive satellite-download GPS receiver for wildlife: field trial on black swans. Wildlife Research. 2017;44(7):558–64. 10.1071/wr17064

[26] Matthews A, Ruykys L, Ellis B, FitzGibbon S, Lunney D, Crowther MS, et al The success of GPS collar deployments on mammals in Australia. Australian Mammalogy. 2013;35(1):65–83. 10.1071/am12021

[27] Merrill SB, Adams LG, Nelson ME, Mech LD. Testing releasable GPS radiocollars on wolves and white-tailed deer. Wildlife Society Bulletin. 1998:830–5.

