# Supplementary material for: Biotelemetry marches on: A cost-effective GPS device for monitoring terrestrial wildlife
Source: PLoS One. 2018 Jul 31;13(7):e0199617. doi: 10.1371/journal.pone.0199617 (PMC6067714; doi:10.1371/journal.pone.0199617)
Supplement: S1 Table — Total cost of GPS wildlife tracker per unit excluding postage. (DOCX) [file pone.0199617.s001.docx]

| Item | Source | USD |
| --- | --- | --- |
| *Wildlife tracker* |  |  |
| GPS device | UniTrack | 175.00 |
| Battery | Master Instruments | 38.21 |
|  |  |  |
| *Firmware* |  |  |
| Website development | Kean Electronics | 73.72 |
| Device firmware update | UniTrack | 60.00 |
|  |  |  |
| *Remote data send function* |  |  |
| HSPA subscription | M2M one | 7.60 |
|  |  |  |
| *Encasing and collar* |  |  |
| Mould | Dalchem | 1.61 |
| Plasticast | Dalchem | 3.26 |
| Lid attachment - screws | Hardware shop | 0.76 |
| Lid attachment - spacers | Small Parts and Bearings | 3.34 |
| Housing attachment - bolts | Hardware shop |  |
| Housing attachment – washers | Hardware shop |  |
| Collar material | Arnold Webbing | 0.30 |
| Collar attachment - bolts | Hardware shop |  |
| Collar attachment - washers | Hardware shop |  |
| Collar attachment - nuts | Hardware shop |  |
| Liquid Electrical Tape | Ebay | 0.94 |
| Conformal Coating | Ebay | 0.90 |
| Epoxy Putty | Hardware shop | 1.06 |
| Foam | Clark Rubber | 0.11 |
| Total |  | **366.81** |
| Consultancy/Labour |  | 300.00 |
| Total including consultancy/labour |  | **666.81** |
