# Supplementary material for: Biotelemetry marches on: A cost-effective GPS device for monitoring terrestrial wildlife
Source: PLoS One. 2018 Jul 31;13(7):e0199617. doi: 10.1371/journal.pone.0199617 (PMC6067714; doi:10.1371/journal.pone.0199617)
Supplement: S1 Appendix — (DOCX) [file pone.0199617.s003.docx]

**Appendix 1.** **Comparing spatially-matched GPS data from stationary and animal-born devices**

**Study location and design**

In this pilot study, we compared HDOP and CSQ values from stationary devices to the same variables from devices attached to free-ranging swamp wallabies (*Wallabia bicolor*). We collected data during February and March 2016 within remnant bushland on Phillip Island. Based on previously collected movement data from several wallabies living in the area, we placed ten stationary devices on a 500 x 500 m grid, consisting of 100 sampling locations, 50 m apart. We randomly allocated each device to a sampling location and shifted it to another location every 24 h until all 100 locations were sampled, over 10 consecutive days. We scheduled the devices to record a fix every 30 min, and averaged the resulting HDOP and CSQ values collected at each location.

We captured two wallabies within the sampling area using double-walled traps [1] baited with carrots and set at dusk. We sedated captured animals with 0.5 mg/kg of Zoletil 100 (Virbac Australia, Sydney) and fitted them with a collar housing our device. We scheduled devices to record a fix every 15 min, and left them on the wallabies until the battery fully drained, which was approximately nine days in both cases.

**Data analysis**

For HDOP and CSQ we applied natural neighbour interpolation [2] in ArcMap 10.2.1 to the data from the stationary grid and the wallaby-borne devices to create overlapping raster surfaces with 1 × 1-m pixels. We used the natural neighbour method to account for the irregular spacing of the data from wallabies, as it performs well in such cases [3, 4]. We assessed concordance between HDOP and CSQ values from the stationary grid and wallaby-borne devices by subtracting values from overlapping pixels. For the devices deployed on wallabies, we could download HDOP values remotely, whereas manual plug-in of the device was required to retrieve CSQ values. We recaptured only one of the two wallabies, so obtained CSQ values from only one wallaby-borne device. We plotted HDOP and CSQ maps, as well as difference maps, and showed values from each pixel and differences between pixel values as histograms. We excluded GPS fixes with no X,Y coordinates, and HDOP and CSQ values of 99 (indicating an error), from the total dataset (grid: n = 370, 8.0%; trackers: n = 69, 5.0%), as well as malfunctioning devices that recorded less than two successful fixes (grid: n = 4, 4%).

**Results**

CSQ values from stationary devices and the device deployed on a wallaby (n = 648) ranged from 0.12 to 16.33 (mean: 6.44 ± SD 2.55) and 0 to 22.30 (mean: 5.15 ± SD 3.91) respectively. As shown in differences between the two maps ranged from -10.56 to 12.23 (mean 0.94 ± SD 2.00) (Fig S1*a and b (iii)*. A large proportion of the CSQ values between the two maps were similar, represented as grey areas in the map and areas around 0 in the histogram, respectively (Fig 1*a and b (iii))*.

The interpolated HDOP values of the stationary test ranged from 0.99 (ideal) to 8.03 (moderate) (mean: 1.68 ± SD 0.67), whereas the values retrieved from devices deployed on free-ranging wallabies (n_total_ = 1368 locations) ranged from 0.70 (ideal) to 29.33 (poor) (mean: 2.19 ± SD 2.56). The difference between the overlapped area of both maps ranged from -27.34 to 2.25 and the high proportion of similarities are shown in grey areas in the map and values around 0 in the histogram (mean 0.50 ± SD 2.19) (Fig 1*c and d (iii)*).


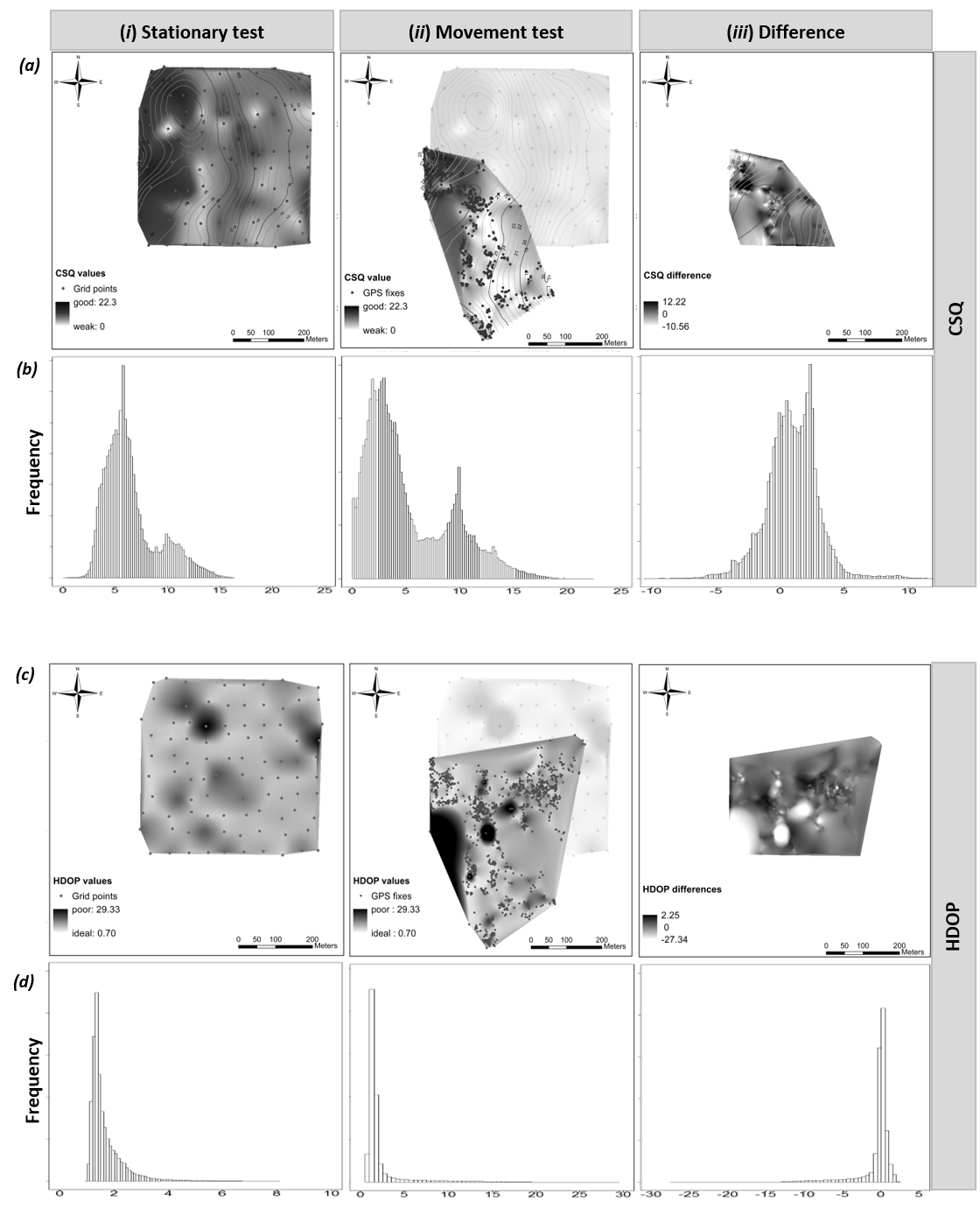


**Fig 1.** **CSQ and HDOP values of stationary test compared to animal deployed devices.** (*a)* and *(c):* Interpolated CSQ and HDOP values of the devices tested in (*i*) a small scale stationary test and (*ii*) compared to devices deployed on free ranging animals. Scales represent the full range of values. (*iii)* Difference between interpolated stationary and movement map. Light grey areas indicate that both maps have similar values within the same area whereas black indicates higher base map values compared to the tracker output and white lower base map values, respectively. *(b) and (d)* Histogram indicating frequency of raster values. (*iii*) Similarities are shown in values around 0.

**References**

1. Di Stefano J, Moyle R, Coulson G. A soft-walled double-layered trap for capture of swamp wallabies *Wallabia bicolor*. Australian Mammalogy. 2005;27(2):235-8. doi: <http://dx.doi.org/10.1071/AM05235>.

2. Sibson R. A brief description of natural neighbor interpolation. Interpreting multivariate data. 1981:21-36.

3. Niebuhr BB, Wosniack ME, Santos MC, Raposo EP, Viswanathan GM, da Luz MG, et al. Survival in patchy landscapes: the interplay between dispersal, habitat loss and fragmentation. Scientific reports. 2015;5.

4. Niebuhr BB, Wosniack ME, Santos MC, Raposo EP, Viswanathan GM, da Luz MG, et al. Survival in patchy landscapes: the interplay between dispersal, habitat loss and fragmentation. Sci Rep. 2015;5:11898. doi: 10.1038/srep11898. PubMed PMID: 26148488; PubMed Central PMCID: PMCPMC4493700.
